# Impact of Gut–Brain Axis and Probiotics on Alzheimer’s Disease

**DOI:** 10.3390/neurolint17100153

**Published:** 2025-09-24

**Authors:** Raghad Tabaza, Richard E. Hartman

**Affiliations:** Department of Psychology, School of Behavioral Health, Loma Linda University, 11130 Anderson St., Loma Linda, CA 92354, USA; rtabaza@students.llu.edu

**Keywords:** neurodegeneration, microbiota, neuroinflammation

## Abstract

This review explores links between the gut–brain axis, probiotics, and Alzheimer’s disease (AD). Using PRISMA-aligned methods, we examined literature from PubMed, ClinicalTrials.gov, and Google Scholar. Studies show that probiotics may reduce AD symptoms by modulating neuroinflammation, microbial composition, and neurotransmitter signaling. Probiotic strains such as *B. breve* and *L. plantarum* were found to be beneficial in early AD or mild cognitive impairment. Limitations include short intervention periods and strain variability. Clinical guidelines and research recommendations are discussed. Mechanisms involve immune signaling, neurotransmitter synthesis (GABA and serotonin), and modulation of systemic inflammation.

## 1. Aims of Literature Review

This review explores how interactions along the gut–brain axis—particularly through microbial modulation—may affect the development or progression of Alzheimer’s disease (AD). The gut–brain axis involves complex bidirectional communication between the gastrointestinal tract and the central nervous system, but the clinical application of probiotics in neurodegeneration is still emerging. Several recent studies suggest that gut microbiota may influence neuroinflammation and cognitive decline, though findings are variable and require further validation.

This review also explores whether probiotic dietary interventions can influence cognitive decline in AD through their effects on the gut–brain axis. Probiotics are being studied as potentially safe and cost-effective options for symptom management in early-stage AD. Another goal is to consolidate current evidence on the gut–brain axis and its role in neurodegenerative diseases. By identifying consistent findings and gaps in research, this review aims to inform future directions. Given the early stage of this field, the findings may serve as a useful resource for clinicians and researchers rather than providing definitive conclusions.

The current review addresses a major health problem that is on the rise and is expected to contribute to progress in the field. Although there are some drug and non-drug options that may help treat some symptoms of AD, there is still no known cure for AD or even a way to stop or slow down its progression. There is, however, increasing scientific studies and evidence around the role of the gut–brain axis and dietary interventions on AD pathology. Improving the scientific knowledge around the gut–brain axis and using dietary interventions as a means of preventing or slowing the progression of AD may have clinical implications by allowing physicians, clinical psychologists, neuropsychologists, clinicians, and other healthcare professionals to use what would be an evidence-based treatment protocol to help patients with AD incorporate a diet beneficial to targeting their gut–brain axis, and thus alleviating the symptoms of AD. The following section reviews key clinical and preclinical studies investigating the effects of probiotic interventions on cognitive function and Alzheimer’s pathology. While many studies report cognitive and inflammatory improvements, findings are still emerging and must be interpreted with attention to methodological variability and study limitations.

Information presented in this literature review could also be used as a guide to a preventative intervention in this field by targeting individuals susceptible to getting diagnosed with AD as a means of decreasing the chances or slowing down the time of actually developing the disease. Doing so would allow us to use dietary interventions with patients long before they are expected to develop the disease around the age of 65. A growing body of experimental and clinical data confirms a key connection between aging and poor diet in the elderly that may be contributing to the pathogenesis of AD.

## 2. Method

### 2.1. Databases

This literature review is based on English-language articles sourced from Google Scholar, PubMed, APA PsychInfo, and ClinicalTrials.gov databases. These primary search engines were utilized to access relevant articles and journals related to the research topic. Most articles and studies referenced were from the past two decades.

### 2.2. Keywords

Keywords utilized for the present review include the following: Alzheimer’s disease (AD), gut–brain axis (GBA), microbiota, microbiome, probiotics, cognitive decline, cognitive impairment, neurodegeneration, dementia, and the gut–brain connection.

## 3. Clinical Importance of Problem

### 3.1. Introduction to Alzheimer’s Disease

Alzheimer’s disease (AD) is a progressive, irreversible brain disorder and the leading cause of dementia, accounting for up to 80% of all dementia diagnoses [1]. It is characterized by cognitive decline across multiple domains, including memory, language, and executive function, and significantly impacts daily living and caregiver burden [2]. Two hallmark neuropathological features define AD: extracellular amyloid-β (Aβ) plaques and intracellular neurofibrillary tangles (NFTs) composed of hyperphosphorylated tau proteins.

These pathological changes often begin decades before clinical symptoms appear, particularly in memory-associated regions such as the hippocampus and entorhinal cortex [3]. With the number of Americans living with AD projected to more than double by 2050, AD represents a growing public health and economic challenge, underscoring the urgent need for more effective and accessible interventions.

AD is a brain disorder that slowly destroys memory and thinking skills, and eventually leads to the destruction of a person’s ability to carry out even the simplest tasks. In the majority of people suffering from AD, symptoms first appear in the mid-60s for those with the late-onset type and, in more rare cases, between the 30s and mid-60s in those with early-onset AD. It has become more clear recently that AD begins decades before the onset of any clinical symptoms of dementia through the accumulation of pathological hallmarks of the disease consisting of Aβ deposits and NFTs [3]. These plaques and tangles are considered the main neuropathological features of AD. Damage initially occurs in parts of the brain involved in memory, including the entorhinal cortex and hippocampus. Later on, it affects areas in the cerebral cortex, such as those responsible for language, reasoning, and social behavior.

### 3.2. Stages of Alzheimer’s Disease

AD progression is split up into three stages [4,5]. The first stage is known as preclinical AD, which precedes any clinical changes. It is defined by measurable changes in biomarkers and poor performance on challenging cognitive tests. The biomarkers may be detectable by brain imaging or by molecular changes in CSF. Currently, there are no known clinical criteria to make a diagnosis of preclinical AD. The second stage of AD is known as mild cognitive impairment (MCI) due to AD. This stage is characterized by the first clinical changes coming to the surface. Mild changes in memory and other cognitive abilities that are noticeable to patients and families may be detected through careful evaluation but are not sufficient enough to interfere with day-to-day activities [6]. In other words, MCI refers to a state of cognitive deterioration that precedes the clinical diagnosis of dementia, as symptoms do not yet compromise an individual’s daily functioning [7]. The third and final stage of AD is dementia, which is characterized by severe impairments in memory, cognition, and motor function, resulting in decreased ability to independently perform functional activities that affect the quality of daily life [8].

Below is a model of the clinical course of AD [9]. The model demonstrates how preclinical AD precedes MCI, which precedes dementia. It is important to note that this diagram represents a hypothetical model for the pathological–clinical continuum of AD but does not necessarily imply that all individuals with biomarker evidence of the AD pathophysiological process will progress to the clinical phases of the illness [9].

### 3.3. Diagnosing Alzheimer’s Disease

Although clinical and neuropsychological measures can be extremely helpful in the detection of AD, a definitive diagnosis of AD requires post-mortem evaluation of brain tissue to observe the two major neuropathological biomarkers. There are currently three autosomal dominant gene mutations known to cause early onset/familial AD: the amyloid precursor protein (APP) gene, and the presenilin 1 and 2 genes. Mutations on these genes are associated with increased β-amyloid production and/or accumulation [10].

Other biomarkers (e.g., biochemical and neuroimaging) may also provide additional information [10,11]. For instance, biochemical biomarkers include those specific to the AD disease process, as well as “nonspecific” biomarkers. Biomarkers specific to the AD disease process (e.g., Aβ levels in cerebrospinal fluid (CSF)) can be used as diagnostic and prognostic markers, whereas nonspecific biomarkers are ones that measure an epiphenomenon of the AD process, such as inflammation or oxidative stress, which could be used to monitor the disease progression and response to treatment [10].

Neuroimaging for early AD diagnosis in living patients may include structural and/or functional imaging [5,10]. Structural imaging includes computer-assisted tomography (CT) and magnetic resonance imaging (MRI). Both modalities provide similar information related to the loss of synapses and neurons associated with AD-related atrophy. Functional neuroimaging includes single-photon emission tomography (SPECT) and positron emission tomography (PET), both which can detect functional differences caused by AD-related neuropathology. Additionally, an amyloid-imaging positron emission tomography (PET) tracer, termed Pittsburgh Compound-B (PiB), can be used in PET scans to image beta-amyloid plaques in neuronal tissues [12]. Klunk and colleagues conducted the first human trial using PiB imaging, involving 16 individuals with mild AD and 9 healthy controls. Compared to the control group, patients with AD showed significantly greater PiB uptake in association cortices, regions typically linked to amyloid accumulation in AD. Within the AD group, the highest levels of PiB retention appeared in the frontal cortex (*p* = 0.0001). Elevated uptake was also detected in the parietal (*p* = 0.0002), temporal (*p* = 0.002), and occipital (*p* = 0.002) cortices, as well as the striatum (*p* = 0.0001). Overall, the results suggest that PET imaging with the novel tracer, PIB, can provide quantitative information on amyloid deposits in living subjects [12].

### 3.4. Treatment of Alzheimer’s Disease—Pharmaceutical

Currently, there is no cure for AD. The six medications currently approved for the treatment of AD target the accumulation of Aβ plaques with a monoclonal antibody (aducanumab/Aduhelm), prevent the breakdown of acetylcholine in the brain’s synapses by inhibiting cholinesterase (donepezil/Aricept, rivastigmine/Exelon, and galantamine/Razadine), regulate glutamate by blocking NMDA receptors (memantine/Namenda), or combine donepezil with memantine (Namzeric).

Currently, three cholinesterase inhibitors (CIs)—donepezil, rivastigmine, and galantamine—are approved for the management of mild to moderate Alzheimer’s disease [13,14]. These medications are considered the primary and standard first-line therapy for AD [15]. Evidence from systematic reviews has shown that all three agents provide benefits for cognitive performance, daily functioning, and overall global outcomes in patients with mild-to-moderate disease [13,16]. No clear differences in effectiveness have been observed among the individual CIs. For patients with moderate-to-severe AD, an additional option is memantine, a noncompetitive antagonist of the N-methyl-D-aspartate receptor [17]. Memantine also exhibits dopamine agonist activity and has been approved for use in patients at this stage of the disease, particularly those experiencing impairments in attention and alertness [18]. Research on disease-modifying therapies for Alzheimer’s disease is ongoing, with efforts focused on targeting the key pathological mechanisms underlying symptoms. These include amyloid-β plaque buildup; neurofibrillary tangle formation; and processes such as inflammation, oxidative stress, iron imbalance, and cholesterol dysregulation [15].

In newly diagnosed Alzheimer’s patients, treatment typically begins with an acetylcholinesterase inhibitor such as donepezil (Aricept), rivastigmine (Exelon), or galantamine (Razadyne), which have proven benefit and good safety (Joe & Ringman, 2019) [19]. Memantine (Namenda) shows little effect in mild AD but provides added cognitive benefit in moderate stages, making combination therapy with cholinesterase inhibitors appropriate for most patients since it is well tolerated.

One of the important pathogenic mechanisms in AD is the chronic inflammation of nerve cells that contribute to the deposition of Aβ. Interestingly, patients with prolonged use of certain nonsteroidal anti-inflammatory (NSAID) drugs such as ibuprofen have been shown to have a reduced risk of developing symptoms of AD [20]. However, long-term use of NSAID is known to have detrimental effects on other areas in the body, such as the kidney and liver.

### 3.5. Treatment of Alzheimer’s Disease—Other

#### 3.5.1. Exercise

Management of cardiovascular risk factors has been found to contribute to overall brain health not just in cerebrovascular disease, but also in neurodegenerative disease [21]. Regular aerobic exercise (flexibility, strength, and agility) was associated with a reduction in the neuropsychiatric symptoms and reduced caregiver burden in patients with AD [22]. In addition to the recommended six months of aerobic exercise, researchers found that irritability, anxiety, apathy, and appetite alterations were the primary improved psychopathological manifestations [22]. In fact, less atrophy was observed in the brains of patients with genetic risk factors for AD who exercised regularly compared with those who did not, suggesting that aerobic activity prevents neurodegeneration [23].

Several non-pharmacological studies have explored the role of exercise in supporting cognitive function in Alzheimer’s disease. One review examined six clinical trials and found that exercise was generally linked to a slower rate of cognitive decline [24]. Additional research has shown that exercise training can delay the progression of cognitive impairment in individuals who are either at risk for or already diagnosed with AD, with aerobic exercise appearing to provide the strongest benefit [25]. Exercise is also thought to exert neuroprotective effects by enhancing brain plasticity and reducing pathological changes. Starting exercise interventions before the onset of clinical symptoms may be especially advantageous, as physical activity stimulates the release of numerous biomolecules from different tissues, some of which contribute to brain health [26]. However, once neurodegeneration has advanced beyond a certain threshold, exercise alone may not be sufficient to reverse or improve symptoms [26]. It is also important to note that many of these studies did not adequately report on race or gender, which limits the generalizability of the findings.

#### 3.5.2. Diet

Recent evidence from systematic reviews indicates that adherence to a Mediterranean diet—characterized by high intake of fruits, vegetables, whole grains, olive oil, legumes, and seafood, along with limited consumption of dairy, poultry, red meat, sweets, and processed foods—is linked to a lower risk of cognitive decline and Alzheimer’s disease [5,27,28]. These diets provide broad metabolic and vascular benefits and may complement targeted probiotic interventions.

Furthermore, ketogenic diets have been shown to improve cerebral glucose metabolism. Another characteristic of AD is known as hypometabolism in the brain, or the decline in cerebral glucose metabolism. This typically occurs before the onset of symptoms but continues as they progress. The brain’s dependance on glucose for fuel puts the brain at risk for declines in cognitive function if that glucose supply is interrupted, such as in the case of hypometabolism. Since patients with AD are known to have a deficit in mitochondrial function and a form of reduced metabolism, more research is needed on hypometabolism [29]. This author suggests that ketone bodies are an efficient alternative fuel for cells that are unable to metabolize glucose and that altering the ketone levels by increasing them indicates cognitive improvements in patients with AD. A more recent article by Jensen et al. [30] details the effects of ketone bodies on brain metabolism in neurodegenerative diseases. They state that ketogenic interventions may ameliorate the energy crisis in neurodegenerative diseases, which are characterized by the deterioration of the brain’s glucose metabolism, providing a therapeutic advantage.

Herbal medicines have been found to be helpful in ameliorating AD pathology and/or progression. Past studies mentioned by Santos-Neto et al. [31] suggest that five main herbs have been found to be particularly useful for cognitive impairment of AD: Melissa officinalis (lemon balm); Salvia officinalis (sage); Yi-Gan (a traditional Japanese herbal medicine that functions as a serotonin modulator); ba wei di huang wan (BDW; a traditional Chinese herbal medicine known to improves cognitive and physical functioning in dementia patients); and Ginkgo biloba (maidenhair tree known to be native to China).

Additionally, Hartman et al. [32] conducted a study targeting whether dietary supplementation with pomegranate juice would influence behavior and AD-like pathology in a transgenic mouse model due to the high levels of antioxidant polyphenolic substances contained in the fruit. They found that mice treated with pomegranate juice learned water maze tasks more quickly and swam faster than controls, and they also had significantly less accumulation of soluble Aβ and amyloid deposition in the hippocampus as compared to control mice. Overall, this specific study demonstrated that pomegranate juice decreases amyloid load and improves behavior in a mouse model of AD. Further, Hartman [33] defined phytochemicals as compounds produced by plants and the phenols, terpenes, and organosulfur and detailed the different phytochemicals that are known to be involved in AD pathology as the following: gingko biloba (EGb761 extract), pomegranates, turmeric and curcumin, garlic, nicotine, and other phytochemicals. In a subsequent review of the literature on phytochemicals on AD pathology, it was noted that several bioactive phytochemical compounds, including vitamins (e.g., tocopherols and folic acid) and other organic compounds (e.g., phenols, terpenes, and organosulfurs), can affect aspects of the AD disease process [34].

Furthermore, researchers studied the importance of curcumin, a natural anti-inflammatory compound and main active ingredient in turmeric, in the prevention of AD pathogenesis [35]. See Table 1 for a summary of relevant findings. Considering that the risk of AD is reduced with increased antioxidant and anti-inflammatory consumption, the phenolic yellow curry pigment curcumin has potent anti-inflammatory and antioxidant activities and can suppress oxidative damage, inflammation, cognitive deficits, and amyloid accumulation. Through mice, researchers were able to demonstrate that curcumin directly binds small beta-amyloid species to block aggregation and fibril formation in vitro and in vivo and concluded that data suggest that low dose curcumin effectively disaggregates amyloid beta, supporting the rationale for curcumin use in clinical trials preventing or treating AD [35]. The mechanisms of curcumin in AD pathology were studied and found that while it decreased beta-amyloid plaques, delayed degradation of neurons, and improved the overall memory in patients with AD, it was also found to be anti-inflammatory and antioxidant [20].

## 4. Gut–Brain Axis

The gut–brain axis (GBA) is a bidirectional communication system between microorganisms residing in the gastrointestinal (GI) tract and the brain [36]. The GI tract is a series of hollow organs that food and liquid travel though when they are swallowed, digested, and eventually leave the body as feces. The enteric nervous system (ENS) is intrinsic to the GI tract and plays an essential role in normal intestinal function, including motility and secretion [37]. It consists of sensory neurons, motor neurons, and interneurons embedded in the wall of the gastrointestinal system. In simple terms, it is considered the nervous system of our gut. Although the ENS may be influenced by the central nervous system (CNS), it can also function independently of both the sympathetic and parasympathetic nervous systems. Recently, the field of brain–gut interactions has expanded and received growing interest with the recognition of complex cross-talk between gut microbiota alterations and brain disorders, including neurodegenerative and psychiatric illnesses, inflammatory, and eating disorders [38]. The primary role of the GBA is to monitor and integrate gut intestinal function with emotional and cognitive brain centers via neuro-immuno-endocrine mediators [36].

The gastrointestinal tract interacts with the central nervous system through the gut–brain axis, which plays a key role in neuronal development and maintenance. When this balance is disrupted, or gut dysbiosis occurs, it can contribute to neurological disorders [39]. Dysbiosis is defined as an imbalance in microbial populations that sends harmful signals to the brain, resulting in low-grade inflammation, oxidative stress, altered energy regulation, and greater cellular degeneration [40]. Research has identified three primary communication pathways between the gut and the brain: neural signaling through afferent and efferent connections, hormonal signaling via gut-derived hormones, and immune signaling mediated by cytokines [41,42]. Together, these mechanisms form a complex communication system that connects systemic imbalances to neurodegenerative processes, influencing insulin regulation, lipid metabolism, oxidative stress, and immune responses [39].

## 5. Gut Microbiome

The microbiome is a diverse population of microbes (bacteria) that live in the GI tract. The GI tract is home to various microorganisms collectively termed the gut microbiome [43]. The terms microbiota and microbiome tend to be used interchangeably in studies. More specifically, the microbiota is defined as the microbial taxa associated with complex organisms such as humans, whereas the microbiome is the catalogue of these microbes and their genes [44]. When it comes to aging, there is an alteration in microbiota, as well as age-associated shifts in the gut microbiome, that contributes to increased predisposition to certain diseases, such as cardiovascular disease, cancer, diabetes, neurodegenerative diseases, and others [45]. In other words, the gut microbiota diversity is perturbed with an increase in pathogenic bacteria at the expense of beneficial ones [46]. The microbiome consists of microbes that are both helpful and potentially harmful. Most are symbiotic (where both the human body and microbiota benefit), and some, in smaller numbers, are pathogenic, meaning they promote disease. According to Harvard School of Public Health [47], pathogenic and symbiotic microbiota coexist without problems in a healthy body. However, if there is a disturbance in that balance (e.g., due to infectious illnesses, certain diets, or the prolonged use of antibiotics or other bacteria-destroying medications), dysbiosis occurs and subsequently stops these normal interactions. As a result, the body may become more susceptible to disease.

Before getting into more details regarding probiotics, it is important to be familiar with a few terms involved in probiotic and gut–microbiome studies. As already mentioned, the gut microbiota is composed of several species of microorganisms, including bacteria, yeast, and viruses. Taxonomically, bacteria are classified according to phyla, classes, orders, families, genera, and species [48]. The dominant gut microbial phyla are Firmicutes, Bacteroidetes, Actinobacteria, Proteobacteria, Fusobacteria, and Verrucomicrobia, with the two phyla, Firmicutes and Bacteroidetes, representing 90% of gut microbiota [48]. One of the ways to classify bacteria is based on the cell membrane: Gram-positive bacteria and Gram-negative bacteria. If a bacterium has a thick, mesh-like membrane called peptidoglycan, it is known as Gram-positive, and if the peptidoglycan layer is thin, it is classified as Gram-negative, which is more difficult to kill due to its hard, protective outer shell [49].

Probiotics are live microorganisms that naturally live in the body. They are considered to be the good or “friendly” bacteria that the body needs and uses to maintain good overall health. There are many different forms of probiotics, including supplements or those found in foods naturally rich in probiotics. Probiotic supplements and foods rich in probiotics contain live microorganisms intended to maintain or improve our “good” bacteria. Probiotic biotherapies are known to create a healthy gut environment by balancing bacterial populations and promoting their favorable metabolic action [39]. Some of the foods that are naturally high in probiotics include yogurt, sauerkraut, pickles, tempeh, aged cheese, and miso. In terms of probiotic supplements, there are various kinds that are marketed to be sold over the counter at most drugstores. Probiotics have been found to play an extremely important role in the gut–brain axis. Probiotics have different strains, or subtypes, of species which function in different ways in the body. The probiotic strain names are often listed on the bottle label of probiotic supplements. Certain strains of probiotics are found to be more beneficial than others, including Lactobacillus and Bifidobacterium.

Probiotics are live microbial supplements that benefit the host by promoting a healthier balance of intestinal microbiota, and they have long been used to alter the composition of the colonic flora [50]. Prebiotics, by contrast, are nondigestible food ingredients that support health by selectively stimulating the growth or activity of specific bacteria already present in the colon. Probiotics are associated with multiple health benefits, including aiding digestion, supporting gut function, and contributing to brain health [51]. As early as 1908, Russian zoologist Élie Metchnikoff, who later won the Nobel Prize for his discovery of phagocytosis, promoted yogurt consumption as a “health food” [52]. A century later, research began to establish that probiotics could also affect central nervous system function through the microbiota–gut–brain axis. Despite this growing recognition, clinical studies evaluating probiotic use in elderly patients with dementia remain limited [53].

Previous studies have shown that probiotic bacteria may ameliorate symptoms of AD by modulating inflammatory reactions driven by beta amyloid deposition. A more recent study showed that certain bacterial products of the intestinal microbiota are correlated with the quantity of amyloid plaques in the brain [54]. The literature provides some studies that have looked at targeting the microbiome by using probiotic interventions as a useful preventative measure for AD.

Westfall and colleagues [39] emphasized that aging is a major factor in the development of neurodegenerative diseases, as it is associated with reduced neurotransmitter activity, persistent inflammation, oxidative stress, and apoptosis—processes that worsen disease progression. Probiotics have been shown to counteract many of these harmful age-related changes. In Alzheimer’s disease, gastrointestinal comorbidities are common, and regulating the gut microbiota has been proposed as a strategy to prevent or lessen symptoms. Diet is another important determinant of gut microbiome composition. Even short-term dietary changes, such as consuming animal- versus plant-based products, can significantly alter microbial communities and influence gene expression [55]. Evidence also indicates that the gut microbiota can impact brain function and behavior, including emotional regulation and related neural pathways [56].

In two different studies, authors concluded that the mode of delivery during childbirth affects the initial microbiome and gut microbiota. Infants who were delivered vaginally had higher amounts of bacteria in their guts compared to infants delivered via Cesarean section [57,58]. Another study found that gut microbiota composition correlates with diet and health in the elderly [59]. Fecal samples were derived from 178 elderly subjects residing in the community, day-hospital, rehabilitation, and long-term residential care. These scientists discovered that the individual microbiota of people in long-stay care was significantly less diverse than that of community residents. Overall, the data from that study support a relationship between diet, microbiota, and health status.

The gut microbiome plays a crucial role in the CNS because the CNS can react to signals from the ENS. This means that our CNS and gut are interconnected and frequently communicate together due to that existing connection between them. Studying the gut–brain axis is relevant to this proposed experiment because a healthy gut is often correlated with a healthy brain. Thus, another way of targeting neurodegenerative diseases would be performed by targeting the gut. The relationship between the gut and brain can have an indirect influence on many factors such as neurotransmitters, stress, anxiety, mood, and behavior. There are two main neurotransmitters involved in the gut–brain axis: serotonin and GABA. A serotonin dysfunction in the GI system could result in impairments in brain function, such as those involved in mood, sleep, and behavior. GABA is a neurotransmitter involved in GI function, and its main purpose is to mediate the ENS. Low levels of GABA are linked to depression and mood disorders [60]. Research suggests that aging alters the gut microbial population, which not only leads to GI disturbances, but also causes CNS disorders such as dementia [61]. Likewise, there is experimental evidence of dysbiosis in AD models and patients contributing to neuroinflammation, cerebrovascular alterations, and amyloid-β formation and thereby influencing the pathophysiology and progression of AD [62]. An emerging area of research suggests that altering the gut microbiome may exert neuroprotective effects similar to other lifestyle interventions [63]. Altering the gut microbiome has been found to target physiological processes associated with dementia risk, and it may influence gut–brain–microbiome axis signaling and impact cognitive functioning. Sanborn and Gunstad [63] share that the gut microbiome can be altered by several means, including disease, diet, prebiotics, probiotics, and physical exercise.

## 6. Gut–Brain Axis and Alzheimer’s Disease

Much of the current understanding of the gut–brain axis has been derived from animal studies. For instance, administration of *Lactobacillus rhamnosus* (JB-1) to mice was shown to reduce stress-related corticosterone levels and anxiety-like behaviors [64]. Research using germ-free animals, or those exposed to antibiotics, probiotics, pathogenic microbes, or fecal microbiota transplants, further suggests that the gut microbiota is involved in Alzheimer’s disease pathology [65]. One investigation found that transgenic AD mice raised in germ-free conditions exhibited lower cerebral amyloid deposition compared to conventionally housed mice [66], supporting the idea that microbial populations influence amyloid accumulation. Using bacterial 16S rRNA sequencing to examine fecal samples from APP transgenic mice, researchers identified marked differences in microbiota composition compared to non-transgenic (wild-type) controls. Generating germ-free APP mice led to a significant reduction in cerebral Aβ pathology relative to mice carrying intestinal microbes. When gut microbiota from conventionally raised APP mice were introduced, Aβ pathology increased, whereas colonization with microbiota from wild-type mice produced a weaker effect. While these findings are informative, differences in immune function and gut ecology between mice and humans limit direct generalization.

Furthermore, an experiment by Kobayashi and peers [67] investigated the effects of oral administration of Bifidobacterium breve strain (*B. breve* A1) on behavior and physiological processes of AD model mice. Researchers reported that giving *B. breve* A1 to Alzheimer’s disease mice improved performance in behavioral tests. The treatment reversed deficits in alternation behavior on the Y maze test and restored latency times in the passive avoidance test, suggesting a protective effect against cognitive decline [67]. They further showed that even non-viable bacterial components or *B. breve* A1’s metabolite, acetate, provided partial benefits in reducing cognitive impairment. Gene expression analysis indicated that *B. breve* A1 consumption suppressed hippocampal inflammatory and immune-related genes that are normally triggered by amyloid-β. In summary, this study showed that giving *B. breve* A1 orally to Alzheimer’s model mice not only alleviated cognitive deficits but also reduced the expression of inflammatory and immune-related genes triggered by Aβ. These findings highlight the potential of *B. breve* A1 as a therapeutic option for preventing cognitive decline in Alzheimer’s disease [67].

A study using *Drosophila melanogaster* (fruit flies) showed that targeting the gut–brain axis improved survival and movement while reducing amyloid-β accumulation and acetylcholinesterase activity [68]. In this model, a symbiotic mixture composed of three probiotic strains—*Lactobacillus plantarum* NCIMB 8826 (Lp8826), *L. fermentum* NCIMB 5221 (Lf5221), and *Bifidobacterium longum* subsp. *infantis* NCIMB 702255 (Bi702255)—together with a polyphenol-rich extract from the herbal tonic Triphala (TFLA), influenced multiple aspects of gut–brain axis signaling. This intervention appeared to protect against the onset of Alzheimer’s disease and slow its progression, potentially through pathways involving peroxisome proliferator-activated receptor gamma (PPARγ). The same study highlighted PPARγ as an important mediator of gut–brain communication and suggested it could serve as a future therapeutic target for probiotic or synbiotic approaches in managing complex chronic diseases such as Alzheimer’s disease [68].

There have been few studies involving humans, indicating the urgency for more clinical work. Some studies have looked at the role of the gut microbiota on brain development, behavior, and/or mood in humans. One clinical trial reported that an altered microbiota can increase production of endotoxins and induce intestinal permeability known as the leaky gut. The leaky gut condition is common in patients with AD. These conditions trigger an inflammatory response that consequently triggers neuroinflammation. This study used omega-3 supplementation to improve the healthy gut microbiota to target the inflammation [69]. Omega-3 fatty acids (FAs) are a group of polyunsaturated fats characterized by a double bond at the third carbon from the terminal end of the chain. The most physiologically relevant omega-3s include eicosapentaenoic acid (EPA), alpha-linolenic acid (ALA), and docosahexaenoic acid (DHA). These fatty acids are critical precursors for neuronal structures, contributing to membrane composition and fluidity, as well as playing roles in signaling, neurotransmission, and regulation of enzymatic activity [69]. Research shows that omega-3 FAs influence the gut microbiome in three primary ways: (1) by modulating microbial composition and abundance, (2) by reducing proinflammatory mediators such as lipopolysaccharides and IL-17, and (3) by regulating levels of short-chain fatty acids and their salts [70].

Research carried out at the Wisconsin Alzheimer’s Disease Research Center compared the intestinal microbiome of patients with Alzheimer’s disease and healthy controls, revealing marked compositional differences. Individuals with AD showed reduced levels of *Firmicutes* and *Actinobacteria*, alongside an increase in *Proteobacteria* and *Bacteroidetes*, which were associated with disease severity [71]. To characterize these microbial shifts, the investigators used bacterial 16S rRNA gene sequencing on DNA extracted from fecal samples of participants with and without an AD diagnosis [71]. They went on to examine the relationship between gut microbiota and AD pathology as measured by CSF biomarkers of AD and found that the gut microbiome of participants with AD demonstrated decreased microbial richness and diversity, with a unique composition compared to control participants.

In a study aimed at differentiating Alzheimer’s disease (AD) from amnestic mild cognitive impairment (MCI), researchers examined 97 participants using RNA sequencing to assess gut bacterial composition, along with cognitive testing through the Mini-Mental State Examination (MMSE), Montreal Cognitive Assessment (MoCA), and Clinical Dementia Rating (CDR) scales [72]. Results showed that AD patients had reduced microbial diversity and distinct microbial profiles compared with controls. Alterations in the gut microbiome were strongly linked to AD, with *Enterobacteriaceae*, a Gram-negative bacterium, associated with both disease presence and progression. Furthermore, levels of pro-inflammatory bacteria, including *Gammaproteobacteria*, *Enterobacteriales*, and *Enterobacteriaceae* within the phylum *Proteobacteria*, rose progressively from healthy individuals to those with MCI and then dementia. These microbial changes were also significantly correlated with clinical severity of AD [72].

Another study performed in China collected fecal samples from 43 AD patients and 43 age- and gender-matched cognitively normal controls. Like other studies, they used the 16S ribosomal RNA sequencing technique to analyze the microbiota composition in feces and found that the gut microbiota composition differed between groups. Various bacteria groups, such as *Bacteroides*, *Actinobacteria*, *Ruminococcus*, *Lachnospiraceae*, and *Selenomonadales*, were found to be variably different in patients with AD compared to control groups: a mild decrease was observed in the abundance of *Bacteroidetes* among AD patients (*p* = 0.039), a slight increase was observed in the abundance of *Actinobacteria*, and *Ruminococcaceae* and *Lachnospiraceae* were among the dominant bacteria. These results led the authors to conclude that the gut microbiota is altered in AD patients and thus may be involved in the pathogenesis of AD [73]. Although the studies performed in China provide relevant findings regarding alterations of gut microbiome in AD individuals, many of the qualitative changes, such as lifestyle, dietary habits, ethnicity, and other comorbidities, were not comparable in China and the USA [74]. Previous experiments and available data confirm the role of gut–microbiota and brain interactions in neurodegeneration and suggest that the inflammatory response, aging, and poor diet in the elderly all contribute to the pathogenesis of AD. Considering the nature of the bidirectionality of the gut–brain axis, neuropathology in the brain may induce microbiota changes, and microbiota changes may induce neuropathology.

New preventative and therapeutic options for AD can be created by changing the gut microbiota through food-based therapy or probiotic supplementation [53]. Researchers have generally accepted that the gut microbiota influences psychological processes such as stress response and cognition [75]. Another study analyzed data collected from fecal samples to compare the gut microbiome of patients with AD versus patients without AD. After analyzing the data, they found that, in AD participants specifically, the gut microbiome has decreased microbial diversity and is different from both age- and sex-matched healthy controls [71].

Diet plays a central role in regulating the gut microbiome, which has downstream effects on the immune system and central nervous system. Imbalances in gut bacterial composition can contribute to gastrointestinal inflammation and have been associated with systemic inflammation and neuroinflammatory processes involved in Alzheimer’s disease [65]. This supports the gut–brain axis as a relevant target for potential therapeutic intervention, although further clinical evidence is required. 

## 7. Probiotics and Alzheimer’s Disease

Scientists have revealed that a daily dose of probiotics, including the strains *Lactobacillus* and *Bifidobacterium* bacteria, taken over a period of 12 weeks is enough to yield moderate, yet significant improvements in the score of elderly Alzheimer’s patients on the MMSE [76]. In this randomized, double-blind clinical trial, 60 participants were assigned to either a control group that consumed milk or a treatment group that received 200 mL/day of probiotic milk containing *Lactobacillus acidophilus*, *Lactobacillus casei*, *Bifidobacterium bifidum*, and *Lactobacillus fermentum* (2 × 10^9^ CFU/g each) for 12 weeks. MMSE scores were collected before and after the intervention, and fasting blood samples were analyzed to measure metabolic changes. Results showed that the probiotic group had a statistically significant improvement in MMSE compared with the control group (*p* < 0.001); however, the magnitude of change was not considered clinically meaningful, since scores still reflected cognitive impairment. Even so, the findings suggest that consistent probiotic consumption can benefit cognitive function and metabolic status in AD patients.

Another study revealed that *L. plantarum* MTCC1325 (Gram-positive strain of probiotic bacteria) might have anti-Alzheimer properties against D-Galactose, a sugar that serves as an energy source, induced in AD [77]. Further studies manipulated the gut microbiota through probiotics oral administration and were able to restore glucose homeostasis in a mouse model of AD [78]. Other experiments have demonstrated that probiotic treatment improves spatial performance and antioxidant biomarkers in the Aβ when administered to animals [79]. This study provides the first proof of the positive effect of probiotics on synaptic plasticity in an animal model of AD. Previous studies have demonstrated that *Lactobacillus plantarum* C29 effectively increases cognitive performance in aged rats and ameliorates scopolamine-induced memory impairment in mice by inhibiting brain inflammation due to excessive lipopolysaccharide in the gut microbiota and restoring hippocampal brain-derived neurotrophic factor (BDNF) expressions [80,81].

A clinical trial by Hwang et al. [82] evaluated the safety and efficacy of *Lactobacillus plantarum* C29-fermented soybean (DW2009) as a dietary supplement for cognitive support. *L. plantarum* C29, originally isolated from kimchi, is recognized for its anti-inflammatory properties. In the study, 100 participants with mild cognitive impairment (MCI) were randomly assigned to receive either DW2009 (800 mg/day, *n* = 50) or placebo (800 mg/day, *n* = 50) for a 12-week period. Cognitive outcomes were assessed using computerized neurocognitive tests focusing on memory and attention, while serum brain-derived neurotrophic factor (BDNF) levels were also measured. Compared with placebo, those receiving DW2009 demonstrated significantly greater improvement in overall cognitive scores (z = 2.36, *p* = 0.02), with the most notable gains in attention (z = 2.34, *p* = 0.02). These cognitive improvements were positively correlated with increased serum BDNF levels following DW2009 intake (t = 2.83, *p* = 0.007). The findings indicate that DW2009 can be safely used to enhance cognitive performance in MCI, and that elevated BDNF levels may underlie these benefits, highlighting the role of the gut–brain axis in addressing cognitive decline. The authors also noted that placebo effects could influence subjective outcomes, underscoring the need for objective biomarkers in future studies.

One of the ways to bridge the existing gap in research is to conduct human intervention studies according to good clinical practices while reporting factors such as active microorganism, dietary components, drugs, or lifestyle that may interfere with probiotic benefits [83]. Clearly, there is evidence on the benefits and importance of probiotics and their effects on different brain and bodily functions. More specifically, additional research is needed on the specific strains that would be the most beneficial for AD symptoms, along with a better understanding of the long-term effects of probiotics.

Given all the research that has been performed on AD, gut–brain axis, and probiotics thus far, there are still many missing gaps in the literature that need to be filled to develop an evidence-based intervention with the goal of treating patients with AD. This current study is expected to broaden the knowledge in this field and contribute to the findings of probiotic supplements, their effects on cognitive symptoms of AD, and the involvement of the gut–brain axis in neurodegenerative disease. The purpose of this study will determine if targeting the gut microbiome would potentially yield to the possibility of improving pathology and symptoms in patients with AD. Scientific findings in this review suggest that probiotics or prebiotics have potential as novel biological safeguards in treatment of AD mainly due to their anti-inflammatory and antioxidant properties. Their ability to improve cognition and metabolic activity, as well as their capacity of producing essential metabolites for gut and brain barrier permeability, seems promising [84].

Kobayashi and colleagues later conducted a randomized, double-blind, placebo-controlled clinical trial, building on findings from earlier mouse studies, to test whether 12 weeks of *Bifidobacterium breve* A1 supplementation could influence cognitive function in older adults with memory complaints. Cognitive performance was evaluated at baseline and after the intervention using the Japanese versions of the Repeatable Battery for the Assessment of Neuropsychological Status (RBANS) and the Mini-Mental State Examination (MMSE). In total, 121 participants were randomized, and 117 completed the study. Individuals in the probiotic group consumed two *B. breve* A1 capsules daily after meals for 12 weeks, while maintaining their usual lifestyle, which was monitored through daily study diaries [85]. At the end of the trial, both groups showed improvements in neuropsychological test scores, with no significant difference between them overall. However, stratified analyses revealed that participants with lower baseline RBANS scores experienced significant gains in the RBANS immediate memory subscale and in MMSE total scores compared to placebo.

The improvement observed by *B. breve* A1 on the immediate memory subscale suggests that this specific probiotic is mainly effective in treating MCI and the early stages of dementia. The study by Kobayashi et al. [85] demonstrated that supplementation with *B. breve* A1 was safe and led to improvements in immediate memory among older adults with subjective memory complaints, particularly those with low baseline RBANS scores. Although the findings are promising, the short intervention period remains a limitation, as longer durations may be necessary to evaluate preventive effects. Compared to MMSE, RBANS provides greater sensitivity in detecting early cognitive changes. The observed cognitive improvements highlight the potential for strain-specific benefits, even when overall group-level effects remain modest.

Another randomized, double-blind, placebo-controlled trail led by researcher Xiao et al. [86] in Japan aimed to test the ability of the probiotic strain *Bifidobacterium breve* (*B. breve*) A1 (MCC1274) to restore cognition in a physically healthy, suspected MCI population. Researchers chose 80 healthy older adults suffering from MCI and divided them into two groups: one group to receive probiotic *B. breve* once daily and the other to receive a placebo for 16 weeks, using a computer-generated algorithm. To assess their cognitive functions, the scientists used the Repeatable Battery for the Assessment of Neuropsychological Status (RBANS) and the Japanese version of the MCI Screen (JMCIS) tests before and after the study. After 16 weeks of supplementation, participants in the probiotic group showed significantly higher RBANS total scores compared with placebo (mean between-group difference = 11.3, 95% CI: 6.7–15.8; *p* < 0.0001). Notable gains were observed in immediate memory, visuospatial/constructional ability, and delayed memory domains (*p* < 0.0001), consistent across both intention-to-treat (ITT) and per-protocol (PP) analyses. The JMCIS score also improved in the probiotic group relative to placebo, approaching significance in ITT analysis (*p* = 0.052) and reaching significance in PP analysis (*p* = 0.036). These findings suggest that *Bifidobacterium breve* A1 is a safe and effective intervention for enhancing memory function in individuals with suspected mild cognitive impairment [86].

An additional study by Ton et al. [87] examined the potential benefits of kefir supplementation on cognitive performance, inflammatory markers, and oxidative stress in elderly patients with Alzheimer’s disease. The researchers proposed that regular consumption of milk fermented with kefir grains might help improve cognitive, metabolic, and cellular impairments in AD. In this uncontrolled clinical trial, participants received probiotic-fermented milk (2 mL/kg daily) for 90 days. Cognitive testing, cytokine expression, oxidative stress markers, and blood cell damage biomarkers were measured at baseline (T0) and after supplementation (T90). Following the intervention, patients demonstrated significant improvements on eight standard cognitive tests, particularly in memory, visuospatial and abstraction abilities, and executive/language function. Cytometric analyses revealed decreases in several inflammatory cytokines and oxidative stress markers, accompanied by a complete (100%) increase in nitric oxide bioavailability. These results indicated that kefir supplementation improved cognitive deficits (*p* < 0.05), likely by modulating systemic inflammation, oxidative stress, and blood cell damage. Consistent with other findings, the study suggested that probiotics may serve as a valuable adjunct therapy for slowing the progression of AD.

Sanborn and colleagues [88] conducted a double-blind, placebo-controlled randomized clinical trial to investigate the effects of *Lactobacillus rhamnosus* GG on cognitive functioning in middle-aged and older adults. The study enrolled 200 participants between the ages of 52 and 75, who were randomly assigned to receive either a daily probiotic or placebo for three months. Cognitive performance was assessed using the NIH Toolbox Total Cognition Score, along with other statistical comparisons, including *t*-tests, chi-squared tests, and repeated-measures ANOVAs. Primary analyses included 145 participants (77 probiotic; 68 placebo), and cognitive impairment was defined based on established criteria (≥1 subtest t-score ≤ 35). Results showed that individuals with cognitive impairment who received the probiotic experienced greater improvements in total cognition scores compared with impaired individuals in the placebo group, as well as cognitively intact participants in either group. The findings indicate that supplementation with *L. rhamnosus* GG may enhance cognitive outcomes in older adults with impairment and support its potential role as a novel approach to maintaining cognitive health during aging [88]. This current study was limited in several ways: slight difference in the duration of supplementation, possible practice effects of NIH Toolbox, and insufficient data regarding changes in the gut microbiome through DNA sequencing.

**Table 1:** Summary of key clinical trials evaluating probiotic interventions in cognitive impairment and Alzheimer’s disease.

Study	Design	Sample Size	Intervention and Duration	Outcomes	Limitations
[35] Yang et al. (2005)	Rodent experimental model	Mice	Microbial manipulation	Modulated amyloid deposition	Animal data, unknown human translation
[68] Westfall et al. (2019)	Review + preclinical	Multiple studies	Probiotics + polyphenols	Neuroprotection, ↓ oxidative stress	Not a clinical trial
[71] Vogt et al. (2017)	Case–control (human postmortem)	25 AD vs. 25 controls	Gut microbiota analysis	Altered diversity, ↑ proinflammatory taxa	Small sample, postmortem only
[76] Akbari et al. (2016)	Randomized, double-blind, placebo-controlled	60 AD patients	Probiotic milk (200 mL/day) for 12 weeks	Improved MMSE, glucose, lipids	Small sample, short duration
[82] Hwang et al. (2019)	Randomized controlled trial	79 elderly	*L. plantarum* C29-fermented soybean, 12 weeks	Improved RBANS	Diet-specific, cultural influence
[85] Kobayashi et al. (2019)	Randomized, placebo-controlled	130 elderly	*B. breve* A1, 20 weeks	Improved RBANS memory	No biomarker analysis
[86] Xiao et al. (2020)	Randomized, double-blind, placebo-controlled	100 MCI	*B. breve* MCC1274, 24 weeks	Improved memory and exec function	Single strain only
[89] Tamtaji et al. (2019)	Randomized, double-blind, placebo-controlled	60 MCI patients	Probiotic + selenium, 12 weeks	Improved MMSE, ↓ inflammation	Confounding: combined treatment
[39] Westfall et al. (2017)	Animal model + review	Rodents	*Lactobacillus* and *Bifidobacterium* strains	Improved cognition, ↓ inflammation	Preclinical, lacks human trial validation

## 8. Related Topics

When it comes to the impact of the gut–brain axis and probiotics on AD, many subtopics fall under this literature review topic, including other dietary interventions, psychological disorders, and other neurodegenerative diseases. Exploring additional dietary interventions to probiotics will be important for the purposes of this literature review. Studies showed that gut-derived inflammatory response with aging and poor diet in the elderly contributes to the pathogenesis of AD. Thus, we will further explore what a healthy diet consists of for the purposes of AD.

According to the Alzheimer’s Association, adopting a heart-healthy diet benefits both the body and brain, and generally consists of foods with low saturated fats. The two main diets that are mentioned when it comes to AD are the Dietary Approaches to Stop Hypertension (DASH) and the Mediterranean diet. As a general rule to follow, both of these diets can help reduce heart disease and therefore may also be able to reduce risks for dementia. The DASH diet consists of foods that are low in saturated fat, total fat, and cholesterol; and high in fruits, vegetables, and low-fat dairy [90]. Furthermore, the Mediterranean diet incorporates different principles of healthy eating that are typically found in the areas bordering the Mediterranean Sea. More specifically, this diet focuses on fruit, vegetables, nuts, and grains, while replacing butter with healthy fats such as olive oil. The Mediterranean diet also limits the consumption of red meat and replaces it with fish and poultry at least twice a week. Instead of salt, herbs are recommended to flavor food [90].

As mentioned earlier, Hartman et al. [32] conducted a study targeting whether dietary supplementation with pomegranate juice would influence behavior and AD-like pathology in a transgenic mouse model due to the high levels of antioxidant polyphenolic substances contained in the fruit. They found that mice treated with pomegranate juice learned water maze tasks more quickly and swam faster than controls, and they also had significantly less accumulation of soluble Aβ and amyloid deposition in the hippocampus as compared to control mice. Overall, this specific study demonstrated that pomegranate juice decreases amyloid load and improves behavior in a mouse model of AD [32]. In a subsequent review, Hartman and Ross [34] addressed epidemiological and experimental evidence for the effects and potential mechanisms of several commonly consumed phytochemicals on neuropathology and outcomes of Alzheimer’s disease and suggested that regular consumption of bioactive phytochemicals from a variety of fruits and vegetables mitigates age- and insult-related neuropathology in Alzheimer’s disease. Overall, there is a trend that suggests that dietary factors, such as the DASH/Mediterranean diet and pomegranate/polyphenols, exert their benefits via the gut microbiota through modification of metabolic functions, anti-inflammatory properties, and support of neurogenesis. The benefit is that these dietary factors are cost-effective, practical, and non-pharmacological interventions, so they are often a good starting point in prevention and treatment.

In terms of emotional regulation, the microbiome has a regulatory role on anxiety, mood, cognition, and pain that is exerted via the gut–brain axis [91]. Other psychological disorders, such as depression and anxiety, are also worth mentioning due to the studies performed on the effects that the gut health can have on mental health. Certain neurotransmitters, such as serotonin and GABA, can guide the discussion around the neuroscience behind the effects of the gut on our brains. Probiotics have been used as supplements to other medications or even alternative treatments for anxiety and depression [72]. Animal studies that have looked at the relationship between probiotics and the immune response found that, after treating mice with probiotics containing *Lactobacillus*, the microbiome and HPA axis were restored [92]. Other studies were also able to demonstrate that treatment for as little as two weeks decreased ACTH levels and corticosterone levels in rats, thus demonstrating the suppressive effects that probiotics have on the HPA axis [93]. Similarly, human studies show comparable reductions in anxiety and depressive symptoms. Patients suffering from chronic stress were given a three-week probiotic treatment containing *a milk drink or a placebo which were consumed daily*. Mood and cognition were measured at baseline, after 10 days of consumption, and after 20 days of consumption. Patients rated an overall happier mood on daily analogue scales using six dimensions of mood, including energetic/tired, composed/anxious, elated/depressed, clearheaded/muddled, confident/unsure, and agreeable/angry [94]. Overall, the study concluded that the consumption of the probiotic improved the mood of individuals who started the study with a poor mood.

Furthermore, a 30-day study consisted of healthy volunteers with no previous depressive symptoms who were given either probiotics or antidepressants. Individuals who were given the probiotics showed reduced cortisol levels and improved self-reported psychological effects similar to participants who were administered Diazepam, a commonly used anti-anxiety medication [95]. Another study that led to a discovery of the possibility of utilizing probiotic supplementation to ameliorate or prevent depression offered 20 healthy participants without current mood disorder a four-week probiotic food-supplement intervention, while 20 control participants received an inert placebo for the same period. In the pre- and post-intervention assessment, cognitive reactivity to sad mood was assessed using the revised Leiden index of depression sensitivity scale [96]. Researchers concluded that those who received the probiotic intervention demonstrated significantly reduced overall cognitive reactivity to sad mood and stated that the study provided evidence regarding the intake of probiotics potentially reducing negative thoughts associated with unhappy mood. These studies have determined the importance of a healthy microbiome and how probiotics have been used with patients suffering from anxiety and depression due to the dysbiosis and inflammation in the CNS being linked as a potential cause of mental illness [97].

Alongside Alzheimer’s disease, Parkinson’s disease (PD) is another progressive neurodegenerative disorder, clinically marked by a mask-like facial expression; tremors (typically unilateral at onset); rigidity of the limbs, trunk, or neck; akinesia (reduced voluntary movement); and postural or balance difficulties. These symptoms stem from degeneration within the basal ganglia and a decline in dopamine production. One widely supported hypothesis for PD pathogenesis is the abnormal buildup of α-synuclein (αSYN), a protein found in many cell types, with PD patients showing elevated expression of αSYN at presynaptic terminals and neurite projections [98]. Normally, αSYN regulates presynaptic release of neurotransmitters such as dopamine, but its overexpression and aggregation are implicated in disease progression. Increasing evidence also links disruption of the brain–gut–microbiota axis to PD, with gastrointestinal symptoms often preceding motor impairments. This supports the theory that disease pathology may propagate from the gut to the brain [99]. According to this model, gut dysbiosis, small-intestinal bacterial overgrowth, and enhanced intestinal permeability can overstimulate the innate immune system, triggering systemic inflammation. In addition, activation of enteric neurons and glial cells may contribute to α-synuclein misfolding, further promoting PD progression [99].

The role of gut microbiota in neuroinflammation and motor impairments has been demonstrated in animal models of Parkinson’s disease. Studies showed that the presence of a normal gut microbiome is essential for PD-related motor and brain pathology, as well as for the production of short-chain fatty acids that trigger microglial activation and worsen PD symptoms [91]. When the microbiome was eliminated in these mice, both microglial activation and pathological changes were reduced, providing strong evidence that the gut microbiome directly contributes to PD pathophysiology [100]. Additionally, mice that received fecal transportation from patients with PD exhibited significant impairment of motor functions compared to mice that received fecal samples from healthy controls, providing even stronger evidence for the involvement of the gut microbiome in the pathology of PD.

Other topics that can inform this literature review include research on other brain disorders that can be further explored by understanding the gut–brain axis. This includes other neurodegenerative diseases, autism spectrum disorder, depression, anxiety, and many others. Connecting the research conducted to better understand these other factors on such topics that affect our brain and behavior can be promising in understanding the research on AD as well. Additionally, exploring research behind lifestyle factors that may play a role in altering the gut–brain connection which can potentially lead to a greater likelihood of developing AD may inform this review. Another broader topic that can inform this review is the various effects that the gut microbiome has on the rest of the body. Understanding how the microbiome can affect our body positively and negatively can deepen our understanding of the organ of interest, the brain.

## 9. Discussion and Clinical Recommendations

The purpose of this literature review is to explore factors related to Alzheimer’s disease (AD) pathology, the gut–brain axis, and the potential role of probiotic dietary interventions. While early research, especially in animal models, has shown encouraging signs, there is still a lack of large, well-controlled clinical trials in humans. Because of this, we cannot yet make strong conclusions about how effective probiotics are in improving AD-related symptoms, like memory loss or cognitive decline.

Probiotics are of interest because of their known benefits for gut health that may influence brain function through the gut–brain connection. However, results from human studies are mixed. There is a lot of variation in the strains used, the dosages, the length of treatment, and how outcomes are measured. While probiotics may offer a safe and affordable option, they should be considered a supportive approach—not a proven treatment for AD. Probiotics are generally safe, especially for healthy individuals, but elderly people may face some risks, such as digestive discomfort or, rarely, systemic infections. Clinicians might consider a short-term probiotic course for people with mild cognitive impairment (MCI), ideally as part of a broader care plan that includes dietary and lifestyle changes.

Another goal of this review is to bring together the current evidence on the gut–brain axis and how it might relate to neurodegenerative conditions like AD.

Since human research in this field is still developing, the focus is more on setting the stage for future studies than on drawing firm conclusions. The gut–brain relationship is real, but its effect seems to be more limited in the later stages of AD, where brain damage becomes irreversible.

Current probiotic studies vary a lot in how they are designed, and many are held back by small sample sizes, short durations, or inconsistent outcome measures. Future research should aim to fix these issues by using standardized cognitive assessments; better tracking of gut health; and larger, longer-term trials. While early findings are promising, probiotics should be seen as an experimental, complementary tool—not a replacement for proven strategies, like a healthy diet and regular exercise.

## Data Availability

No new data were created or analyzed in this study.
